# Intravesical Gemcitabine and Docetaxel Therapy for BCG-Naïve Patients: A Promising Approach to Non-Muscle Invasive Bladder Cancer

**DOI:** 10.3390/life14070789

**Published:** 2024-06-22

**Authors:** Mirko Bakula, Tvrtko Hudolin, Nikola Knezevic, Zoran Zimak, Jerko Andelic, Ilija Juric, Marija Gamulin, Milena Gnjidic, Zeljko Kastelan

**Affiliations:** 1 Department of Urology, University Hospital Centre Zagreb, Kispaticeva 12, 10000 Zagreb, Croatia; mirko.bakula@gmail.com (M.B.); knezevicdr@gmail.com (N.K.); zoran.zimak@gmail.com (Z.Z.); jerko.andelic@gmail.com (J.A.); ilija.jur1@gmail.com (I.J.); zeljko.kastelan@gmail.com (Z.K.); 2 School of Medicine, University of Zagreb, Salata 3, 10000 Zagreb, Croatia; mgamulin8@gmail.com; 3 Department of Oncology, University Hospital Centre Zagreb, Kispaticeva 12, 10000 Zagreb, Croatia; milenagnjidic5@gmail.com

**Keywords:** BCG shortage, non-muscle invasive bladder cancer, BCG-naïve, docetaxel, gemcitabine

## Abstract

Bacillus Calmette-Guérin (BCG) therapy for patients with non-muscle invasive bladder cancer (NMIBC) faces limitations in efficacy and significant side effects, aggravated by a recent global shortage. In this prospective clinical study, we report the outcomes of sequential intravesical administration of gemcitabine and docetaxel (Gem/Doce) as a first-line treatment for BCG-naïve patients with high-risk NMIBC (HR NMIBC). From October 2019 until April 2022, we enrolled 52 patients and followed the treatment protocol set forth by the University of Iowa. Follow-up assessments were conducted every 3 months. In this cohort, 25 (48.1%) patients were diagnosed with high-grade T1 (T1HG) bladder cancer, 10 (19.2%) patients had carcinoma in situ (CIS), and 17 (32.7%) patients had a combination of T1HG+CIS. The median time to first recurrence in the T1HG, CIS, and T1HG+CIS groups was 11, 10.5, and 8.8 months, respectively. The recurrence-free survival was 98.1%, 94.2%, and 80.8% at 6, 9, and 12 months, respectively. The rate of progression-free survival was 100%, 98.1%, and 92.3% at 6, 9, and 12 months, respectively. We demonstrated the safety and efficacy of Gem/Doce therapy in BCG-naïve patients with HR NMIBC during a one-year follow-up. Further research with extended follow-ups, as well as direct comparisons of Gem/Doce with other anticancer agents, is essential.

## 1. Introduction

Bladder cancer is a significant public health problem, ranking as the eleventh most prevalent cancer in the world and the fifth most common cancer in the European Union [1]. It is estimated that approximately 550,000 new cases of bladder cancer are diagnosed annually, with a higher prevalence in developed countries [2]. Smoking and occupational exposures such as aromatic amines, polycyclic aromatic hydrocarbons, and benzenes are the strongest established risk factors, but the contributions of other environmental and genetic factors to the development of bladder cancer are increasingly being recognized [3,4]. The development and the progression of bladder cancer are governed by complex molecular processes within bladder cells that control how tumors start, develop, and spread. Changes in important cell signaling pathways, disruptions in the regulation of cell division, and the ability of cancer cells to evade detection by the immune system are crucial factors in the transformation of normal bladder cells into cancerous ones [5]. Most bladder cancers are of urothelial histology, and localized urothelial carcinoma of the bladder is broadly categorized into non-muscle invasive bladder cancer (NMIBC) and muscle-invasive disease. The commonly used Tumor, Node, Metastasis (TNM) staging system defines NMIBC as a noninvasive papillary tumor (Ta), carcinoma in situ (CIS), or with invasion limited to the lamina propria (T1). NMIBC accounts for 70–75% of all bladder cancer cases, and almost 75% of the noninvasive papillary tumors detected are at the Ta stage. Noninvasive papillary lesions or Ta-stage tumors have a low risk of progression to invasive disease, although their biologic behavior is dependent on the grade of the papillary lesion. The remaining 25% imply invasion into the lamina propria or pT1 stage, which tend to be fragile and therefore bleed easily, causing the symptom of hematuria early in the course of the disease. They also tend to recur; 31–78% of these tumors will recur, either at the same stage as the initial tumor or at an advanced stage [6,7]. A flat lesion that extends throughout the mucosal layer without invading the basement membrane is termed CIS according to TNM staging criteria, and all are classified as high grade, thus, warranting investigation for specific genetic mutations and histopathologic characteristics [8]. Upon histopathological examination following the transurethral resection of bladder tumor (TURBT), the lesion typically displays a flat morphology, with cytologic abnormalities throughout the tissue thickness and an intact basement [9,10].

In NMIBC, patient risk is estimated using prognostic models, which allow the stratification of patients into risk groups based on clinical and pathological characteristics. Various models and stratification tables that outline the clinical characteristics of different risk groups have been created by reputable organizations such as the European Association of Urology (EAU) and the American Urological Association (AUA), as well as independent research entities [11,12]. A recent risk model formulated by the EAU aims to predict the time until progression in patients diagnosed with Ta/T1 NMIBC who undergo TURBT followed by intravesical chemotherapy. This model incorporates several predictive factors including age, tumor count, stage, maximum tumor diameter, tumor grade according to either the WHO 1973 or WHO 2004/2016 grading systems, and the presence of CIS [13,14].

The EAU, whose guidelines have been endorsed by more than 50 urological societies and associations, recommends stratification of patients into three prognostic factor risk groups, as follows: low-, intermediate-, and high-risk, which includes a subgroup of the highest risk tumors. Patients with high-risk NMIBC (Ta/T1 with high grade, and/or CIS) represent a challenging group with an increased 5-year risk of recurrence (up to 80%) and progression (up to 50%) according to the EORTC risk stratification tables [11]. Despite advancements in diagnostic and therapeutic options, NMIBC continues to pose a significant therapeutic challenge due to its high recurrence and progression rates [15].

In this study, the focus was on a group of patients with high-risk NMIBC (HR NMIBC). Even after 40 years since its approval, Bacillus Calmette-Guérin (BCG) therapy retains its status as the gold standard for intravesical treatment of NMIBC, despite its inefficacy in 30% to 77% of patients, who may experience disease recurrence within 5 years, and its association with significant side effects [13,16,17,18,19,20]. The global shortage of the BCG vaccine, primarily utilized in the treatment of bladder cancer, has emerged as a significant concern in recent decades. Increasingly, intravesical chemotherapy with gemcitabine/docetaxel (Gem/Doce) has been utilized in many published studies [21,22,23].

In this prospective clinical study, we demonstrated the safety and efficacy of Gem/Doce therapy in BCG-naïve patients with HR NMIBC during a one-year follow-up.

## 2. Materials and Methods

### 2.1. Study Design and Patients

The study was conducted in accordance with the Declaration of Helsinki and approved by the Ethics Committee of the University Hospital Center Zagreb (02/21 AG: 8.1-20/81-2, 8 June 2020). Informed consent was obtained from all participants.

From October 2019 until April 2022, 52 BCG-naïve patients diagnosed with HR NMIBC following complete TURBT were enrolled in this prospective cohort study. Patients were scheduled to undergo a treatment plan consisting of 6 weekly sequential intravesical instillations of 1 g gemcitabine and 37.5 mg docetaxel, followed by monthly maintenance instillations for one or two years for those showing no evidence of high-grade (HG) tumor recurrence.

We collected comprehensive data, including information on sex, age, height, weight, body mass index (BMI), chronic diseases, therapy details, and prior NMIBC treatment history, along with cystoscopic, operative, and pathologic findings, as well as follow-up records.

Risk stratification followed the criteria outlined by the EAU [11]. Patients with a history of prior BCG treatment for any duration were excluded, and so were those who did not undergo the recommended follow-up surveillance during the study period.

### 2.2. Gem/Doce Instillation

The Gem/Doce treatment protocol followed the well-established guidelines set forth by Steinberg et al. at the University of Iowa [23]. After a negative finding of urinary infection (urine dipstick or culture test), induction was sequentially administered intravesically once a week for 6 consecutive weeks. After catheter placement and bladder emptying, slow instillation of one gram of gemcitabine in 50 mL of normal saline solution (NSS) was conducted, followed by clamping the catheter for 90 min.

A catheter was placed into the bladder and the bladder was drained. One gram of gemcitabine in 50 mL of NSS was slowly instilled into the bladder and the catheter was clamped for 90 min. Afterwards, the bladder was emptied, and gradual instillation of 37.5 mg of docetaxel in 50 mL of NSS was administered. Following the instillation, the catheter was removed, and patients were instructed to urinate after 90–120 min. In subsequent instillations, patients were asked about any discomfort, side effects, or changes in their quality of life that may have arisen due to the therapy. In patients without HG recurrence at the initial follow-up after induction, monthly maintenance therapy was initiated.

### 2.3. Surveillance

Follow-up assessments were conducted every 3 months, involving office-based cystoscopy, cytologic examination of urine, and blood tests. The presence of metastatic or synchronous urothelial cancer disease was evaluated using conventional ultrasound, computed tomography, and/or magnetic resonance imaging. All biopsy and cytology findings were evaluated by genitourinary pathologists and cytopathologists for evidence of disease recurrence/progression. If suspicious cystoscopic or imaging findings or positive cytologic results were observed during the follow-up, patients underwent additional procedures including random bladder biopsies, urethral biopsies, bilateral upper tract barbotage cytology tests, and bilateral retrograde pyelograms. Treatment was continued in patients who did not show signs of disease recurrence or showed low-grade recurrence, while it was discontinued in those with HG recurrence. The progression was characterized with tumor advancement in the TNM stage and/or tumor cell differentiation from low-grade to high-grade during the follow-up. High-grade recurrence-free survival (HG RFS) was used to evaluate treatment efficacy. This specifically refers to the length of time during which a patient remains free from any recurrence of high-grade tumors after initial treatment.

### 2.4. Statistical Analysis

Statistical analysis was performed using the STATISTICA 6.1 program (StatSoft Inc., Tulsa, OK, USA). Numerical data were described by descriptive statistics (mean, median, range, standard deviation), and descriptive data by frequency tables. Follow-up data of disease progression and patient survival were analyzed with the Kaplan–Meier method. Comparisons of two or more groups of patients were performed by the χ^2^-test and the log-rank test, respectively. The level of statistical significance was set at 95% (α = 0.05).

## 3. Results

### 3.1. Patient Demographics

This study included 52 patients with HG NMIBC who had not previously received BCG. The mean age of the patients at the time of study inclusion was 69.27 ± 11.45 years. There were 44 (84.6%) male and 8 (15.4%) female patients. The mean BMI was 27.55 ± 5.09, lower urinary tract symptoms were detected in 15 (28.8%) patients, and 14 (26.9%) patients had diabetes mellitus (Table 1).

In this cohort, after final complete TURBT, 25 (48.1%) patients were diagnosed with high-grade T1 (T1HG) bladder cancer, 10 (19.2%) patients had CIS, and 17 (32.7%) patients had a combination of CIS+T1HG (Table 2).

There was a planned delay of at least 20 days in the first weekly induction course of Gem/Doce after TURBT. The mean delay was 1.55 ± 1.07 (0.73–6.57) months (Table 1). In patients with an extreme delay in starting treatment, cystoscopic assessments and radiological diagnostics were performed to verify the state of non-recurrence.

The induction course was completely performed in 50 (96.1%) patients. Of the enrolled patients, 1 had only two induction instillations due to unrelated medical complications, while another patient had a urinary infection, due to which he missed the final instillation needed to complete the induction cycle.

### 3.2. Survival Outcomes

There were ten (19.2%) patients with bladder recurrence during the follow-up. There was no recurrence in the first 4 months of follow-up, with TURBT performed in six patients due to cystoscopy-related suspicion of bladder cancer. In all patient groups, there were fourteen TURBT performed during the follow-up with no bladder cancer diagnosed in the specimens collected, which could be explained by excessive caution for the sake of new therapy initiation. In two (3.8%) patients, at the 11-month follow-up after TURBT, downstaging to TaLG cancer was observed and therapy was continued. There were three (5.8%) cases of progression upon TURBT, which occurred at the 11- and 12-month follow-ups, and one (1.9%) case of radiologically diagnosed progression involving retroperitoneal mass and lymphadenopathy, with no evidence of bladder cancer, at the 9-month follow-up. In two (3.8%) patients with variant histology, no recurrences occurred during the follow-up, with one TURBT performed due to suspected recurrence.

At the time of first recurrence, three (12%) patients were in the T1HG group, two (20%) patients were in the CIS group, and five (29.4) patients were in the T1HG+CIS group. The median times to first recurrence in the T1HG, CIS, and T1HG+CIS groups were 11, 10.5, and 8.8 months, respectively (Table 3).

**Figure 1:**
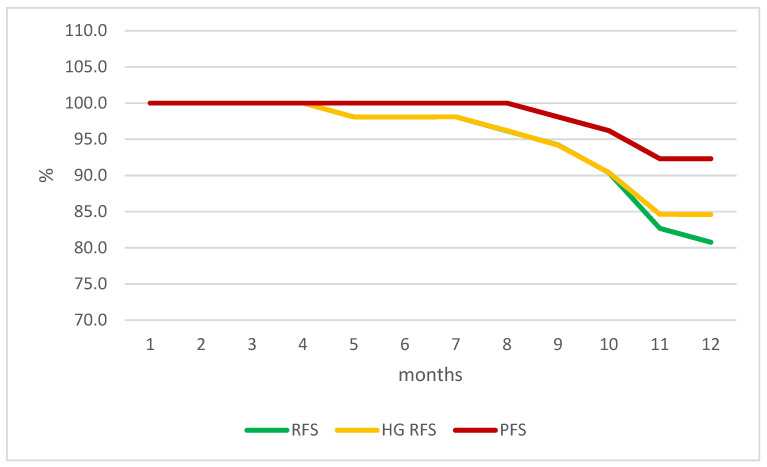
Survival outcomes graph, by months.

The RFS was 98.1%, 94.2%, and 80.8% at 6, 9, and 12 months, respectively. The HG RFS was 98.1%, 94.2%, and 84.6% at 6, 9, and 12 months, respectively. The rate of progression-free survival (PFS) was 100%, 98.1%, and 92.3% at 6, 9, and 12 months, respectively (Table 3, Figure 1).

The χ^2^-test used to test differences among the three groups showed that there was no statistically significant difference between the number of patients with no recurrence and the number from the initial histopathologic diagnosis (*p* = 0.295). The rate of disease recurrence was 12% and occurred at the 11-month follow-up in the T1HG group, whereas in the T1HG+CIS group it was 79.6% and occurred after 5 months (mean, 8.8 months). The log-rank test indicated that there was no statistically significant difference in the number of patients without recurrence between the T1HG and T1HG+CIS histopathology groups (*p* = 0.129).

Regarding the considerable statistical discrepancy between male and female patients, the χ^2^-test showed that there was no statistically significant sex difference, which implies that a considerable statistical discrepancy between male and female patients could not influence the statistical results presented in Table 3 (Table 4). The survival analysis was not verified because it has already been emphasized that the results are not reliable due to the small number of patients.

### 3.3. Progression and Survival

There were no bladder cancer-related deaths during the observed period, and cancer-specific survival was 100% at 12 months. At the 10-month follow-up, one (1.9%) patient had died from a medical condition unrelated to bladder cancer, and overall survival was 98.1%. One patient with TURBT-diagnosed T2 progression underwent radical cystectomy (Table 5). Definitive pathology reported it as pT3bN0. In one patient with radiologically diagnosed progression involving retroperitoneal mass and lymphadenopathy, chemotherapy was initiated.

## 4. Discussion

A team-based approach to managing NMIBC involves healthcare professionals with different specialties working together to provide the best possible care and results for patients. This method acknowledges the intricate nature of NMIBC treatment, which often demands input from urologists, oncologists, radiologists, pathologists, and other specialists. The process begins with ensuring an accurate diagnosis, as well as the staging of NMIBC using procedures such as cystoscopy, imaging scans, and examining tissue samples under a microscope. Urologists lead this initial phase, conducting the necessary tests and coordinating the initial treatment plans. After diagnosis, treatment decisions are tailored to each patient’s specific situation, considering factors such as tumor stage, grade, and risk level. Treatments may include procedures such as reTURBT, intravesical therapy, or radical surgery. Oncologists and urologists collaborate closely to determine the most suitable treatment plan for each patient.

The BCG intravesical treatment involves an initial regimen of 6 weekly administrations, beginning a couple of weeks post-tumor removal. This is then succeeded by a maintenance plan, with extra BCG administrations every 3 to 6 months, spanning 1 to 3 years [24]. Many studies have been published on BCG’s efficacy, and the usual findings are that it induces initial complete response rates of 55–65% for high-risk papillary tumors and 70–75% for CIS [13,24,25]. Maintenance plans vary widely across published research and healthcare institutions, often adapting based on patient tolerance and adherence. These variations have undergone testing in clinical studies, with specific factors explored including the frequency and dosage of BCG instillations [26]. The SWOG 8507 protocol, which involves six instillations over 6 weeks followed by a maintenance regimen of weekly BCG instillations for 3 weeks at 3-month intervals for up to 36 months, has shown superior efficacy in preventing recurrence and progression compared to induction therapy alone. In high-risk patients, a full-dose induction course combined with a 3-year BCG maintenance schedule, following the SWOG 8507 protocol, has led to reduced recurrence rates compared to a 1-year maintenance schedule, although no significant differences have been observed in long-term outcomes related to progression or mortality. In the last decade, partly because of the global BCG shortage, ongoing efforts have aimed to assess the efficacy of reducing the number of BCG instillations during the induction phase, as explored in the NIMBUS trial [27].

However, the global shortage of BCG, primarily attributed to manufacturing challenges and disruptions in vaccine production, coupled with a high global demand, has significantly impacted the availability of this essential treatment option for bladder cancer patients [28,29]. To address these challenges, a range of strategies have been implemented, including the recommendation to reserve BCG for patients at the highest risk of progression. Furthermore, efforts have been made to optimize treatment protocols, with special emphasis on research into novel immunotherapies, chemotherapies, and treatments for bladder cancer. Unfortunately, many studies suffer from notable bias, particularly retrospective ones involving small groups of patients, making it challenging to accurately distinguish between BCG failure and high tumor risk levels, and sometimes this information is not even provided. Despite these limitations, alternative intravesical therapies for NMIBC have long been explored. These include mitomycin C (MMC), valrubicin, gemcitabine, docetaxel, and combination therapies. In studies with BCG-naïve patients receiving MMC, the results demonstrated a 5-year RFS of 34%. MMC monotherapy, applied after an induction course of BCG failed, was found to have a 3-year RFS of 19% [30]. Valrubicin has been studied for the treatment of CIS in patients with previous exposure to BCG. The DFS was 21% at 6 months in a study by Steinberg et al. in which 99% of patients had at least two induction courses [21]. Docetaxel induction provided 1- and 3-year RFS rates of 40% and 25%, respectively, in a BCG failure cohort without maintenance [31]. Intravesical gemcitabine was found to provide RFS rates of 28% at 1 year and 21% at 2 years post-therapy [32]. Among the examined mono- and combination therapies, sequential intravesical administration of Gem/Doce has emerged as a promising therapeutic strategy for patients with NMIBC, and the main aim of our study was to investigate this alternative to BCG therapy.

Gemcitabine and docetaxel are both FDA-approved anti-cancer drugs. The efficacy of combining docetaxel and gemcitabine for treating bladder cancer stems from several factors. Firstly, the cooperative action of these drugs enhances their ability to kill cancer cells, leading to better treatment outcomes. Secondly, using them together may help to overcome the resistance that can develop against single-drug chemotherapy, thus, making the treatment more effective at halting tumor growth. Moreover, this combination therapy is well-tolerated by patients, allowing for longer treatment durations, which in turn improves disease control and patient outcomes.

Gemcitabine and docetaxel were initially introduced in 2014 as an alternative for patients who did not respond to BCG treatment [23]. However, given the ongoing and severe scarcity of BCG within both our and other health systems, and based on the available data at the time, our multidisciplinary team at the institution decided to implement the use of Gem/Doce in a BCG-naïve setting. While conducting therapy, our decision to use Gem/Doce in BCG-naïve patients was validated by the publication of two pivotal papers that emphasize the safety and efficacy of this approach [33,34]. Gemcitabine, a nucleoside analog, and docetaxel, a taxane chemotherapy agent, when administered directly into the bladder, demonstrate localized cytotoxic effects without causing systemic toxicity. The use of this principle involving two or more drugs is justified and has proven effective in other urologic or malignant diseases, thus, it is reasonable to expect that the combination of drugs will yield better results than using either of them alone.

Since 2014, Gem/Doce has mostly been reported as being efficacious and well tolerated in patients who experienced BCG failure, or as a salvage therapy, with 54% one-year treatment success [23]. Catalyzed by the BCG shortage and encouraging initial results, more studies have reported favorable outcomes, including improved disease-free survival, reduced recurrence rates, and acceptable tolerability profiles [35,36].

In this prospective single-arm cohort study, we examined the efficacy of Gem/Doce in BCG-naïve patients with high-risk NMIBC, and examined its use as a maintenance therapy in HG recurrence-free patients during the first checkpoint surveillance after full induction.

Our institutional experience with Gem/Doce therapy in high-risk BCG-naïve patients indicated good efficacy, with RFS rates of 98.1% and 80.8% at 6 and 12 months, respectively. HG RFS was 84.6% and PFS was 92.3% at the 12-month follow-up. During the one-year follow-up, 81% of our patients were free of cancer and 84.6% had HG RFS; slightly better results, including an 87% HG RFS at the 12-month follow-up, have been reported by McElree et al. We had more patients with CIS compared to their study (52% vs. 44%), and they also showed that the majority of first recurrences had a component of CIS, additionally confirming its importance in disease recurrence. Furthermore, they included patients with HG Ta bladder cancer, and all these may be potential explanations for their slightly better results [33].

MeElree et al. published a large study on 312 patients, of which almost 44% had CIS (alone or in combination) and almost a third had a Ta cancer, with HG RFS in 71% of the patients with BCG treatment and 85% of the patients with Gem/Doce [34], which is actually the same result as we found in our study, confirming the efficacy of this therapy especially in older patients who may be increasingly immunosenescent and, therefore, less suitable for BCG treatment. These authors also demonstrated that patients were less likely to quit Gem/Doce treatment because they had a better quality of life and experienced fewer toxic effects compared to BCG [34], confirming the findings reported by other studies [37]. In our study, only one patient reported a—probably unrelated—complication that postponed treatment, thus verifying the safety and tolerability of this treatment.

Daniels et al. evaluated Gem/Doce therapy in a cohort of patients that had a mean of 11.6 intravesical treatments prior to Gem/Doce—with the majority (>80%) of them being BCG, but also including MMC and valrubicin, making this cohort relatively heterogeneous, with the only possible bias related to previous therapy and not to Gem/Doce—and found a HG recurrence rate of only 10.2% at a 24-month median follow-up. In any case, all these data show that Gem/Doce therapy is at least as good as BCG treatment for HG RFS rates, which are closer to 80% [38]. There is also the question of how long Gem/Doce therapy should be administered in order to obtain optimal results? And will prolonged use give our patients even better results, as has been shown with BCG treatment?

As we have shown in the Results section, during the observation period, 14 TURBT were performed in which bladder cancer was not proven. Most of them were performed during the initial follow-up period, which we can attribute to the increased caution towards introducing new therapy into the NMIBC treatment protocol. In such patients, cystoscopic examination showed a solid formation, with a non-papillary appearance. The histopathologic findings mainly indicated an area of necrosis and inflammatory infiltrate, perhaps with anticancer impact [39]. Over the course of the observation period, the number of biopsies that did not indicate recurrence decreased and the number of positive cancer findings increased, which indicated a learning curve for cystoscopic examination of patients undergoing Gem/Doce therapy. Regarding the delay between TURBT and the start of therapy, Zeng et al. reported a typical duration of 4 to 6 weeks in their group of patients [22]. This result is similar to ours, as in our group the mean delay was 1.55 ± 1.07 months, which coincided with our plans and expectations. The extreme delay of initial therapy after TURBT happened in only in two patients. In both patients, as described previously in Results, cystoscopic assessment and radiological diagnostics were performed prior to therapy initiation to verify the state of no recurrence.

Regarding the financial aspect, an induction course of Gem/Doce is roughly 125% of the cost of an induction course of BCG and 1/3 of the cost of MMC induction, with docetaxel making up most of that cost [38]. Valrubicin has been shown to cost $20,000 per induction course [40]. Avoiding cystectomy can be financially beneficial, considering that the average cost of the initial hospital stay for cystectomy patients is $33,202, with additional mean costs of $14,417 for readmissions [41]. Cost reduction—even in small amounts—when dealing with the costliest cancer at a population level, holds significant importance for patients, healthcare providers, and insurance companies alike. This is particularly crucial as the healthcare landscape increasingly emphasizes enhancing the value of care delivered. Bladder cancer, being one of the most expensive cancers to treat, places a substantial financial burden on patients, healthcare systems, and insurers. Therefore, any reduction in the costs associated with treatments, such as avoiding the expense of cystectomy and subsequent readmissions, can have notable benefits across the board.

Regarding the limitations, although our study was prospective, the lack of a control group may be considered a limitation. However, given the global shortage of BCG over an extended period, and the limited efficacy of some other types of intravesical therapy for patients with NMIBC, we chose to administer Gem/Doce to all our patients. After completing the therapy, we intend to present the results recorded in this cohort.

While the initial findings are promising, further research is necessary to refine the dosage and treatment schedule, as well as to identify markers that predict how patients will respond to this therapy. Exploring potential combinations with targeted therapies or immunotherapy agents is also crucial. Ongoing clinical trials are actively investigating the efficacy of Gem/Doce combination therapy across different subtypes of bladder cancer and treatment scenarios. The aim is to solidify its position as a standard treatment option for managing this complex disease.

Further research with extended follow-ups, as well as direct comparisons between Gem/Doce and BCG, or combinations of BCG with other drugs such as mitomycin, is essential in order to unequivocally establish the role of Gem/Doce in NMIBC.

## Figures and Tables

**Table 1:** Patient descriptive statistics.

	Valid N	Mean	Median	Minimum	Maximum	SD
Age (years)	52	69.27	68.20	44.70	93	11.45
BMI	52	27.55	26.78	19.15	41.97	5.09
TURBT to 1st therapy (months)	52	1.55	1.23	0.73	6.57	1.07
Sex	*n*			Percent		
M	44			84.6		
F	8			15.4		
Total	52			100.0		
DM	*n*			Percent		
No	38			73.1		
Yes	14			26.9		
Total	52			100.0		
LUTS	*n*			Percent		
No	37			71.2		
Yes	15			28.8		
Total	52			100.0		

SD—standard deviation; BMI—body mass index; TURBT—transurethral resection of bladder cancer; M—male; F—female; DM—diabetes mellitus; LUTS—lower urinary tract symptoms.

**Table 2:** Histopathology after complete TURBT.

Histopathology	*n*	Percent
T1HG	25	48.1
CIS	10	19.2
T1HG+CIS	17	32.7
Total	52	100.0

T1HG—T1 high-grade; CIS—carcinoma in situ.

**Table 3:** Tumor recurrence.

Histopathology	Recurrence in Total	Median Time to Recurrence (Months)
T1HG	3 (12%)	11
CIS	2 (20%)	10.5
T1HG+CIS	5 (29.4%)	8.8

HG—high grade; CIS—carcinoma in situ.

**Table 4:** Sex discrepancy in survival prognosis.

Two-Way Summary Table: Observed Frequencies				
Sex	T1HG	CIS	T1HG+CIS	Row
Male	21	9	14	44
Column %	84.00%	90.00%	82.35%	
Female	4	1	3	8
Column %	16.00%	10.00%	17.65%	
Total	25	10	17	52
Statistics:				
		Chi-Squared	df	p
Pearson’s Chi-Squared		0.296	2	0.862

T1HG—T1 high-grade; CIS—carcinoma in situ.

**Table 5:** Survival outcomes by months.

Month of Therapy	RFS	HG RFS	PFS
1	100.0	100.0	100.0
2	100.0	100.0	100.0
3	100.0	100.0	100.0
4	100.0	100.0	100.0
5	98.1	98.1	100.0
6	98.1	98.1	100.0
7	98.1	98.1	100.0
8	96.2	96.2	100.0
9	94.2	94.2	98.1
10	90.4	90.4	96.2
11	82.7	84.6	92.3
12	80.8	84.6	92.3

HG—high grade; RFS—recurrence-free survival; PFS—progression-free survival.

## Data Availability

The raw data supporting the conclusions of this article will be made available by the authors upon request.
